# *Smallanthus sonchifolius* as a trap plant for *Bemisia tabaci*: host preference, host suitability, and semi-field control effects under protected tomato cultivation

**DOI:** 10.3389/fpls.2026.1973439

**Published:** 2026-10-07

**Authors:** Huangkai Tang, Yuze An, Yun Gao, Lingling Liu, Songtao Tan, Yana Li, Wei Wang, Binyan Cai, Li Ding, Jiani Liu, Shaowu Yang

**Affiliations:** 1 Yunnan Urban Agricultural Engineering and Technological Research Center, College of Agriculture and Bioscience, Kunming University, Kunming, China; 2 Yuxi Normal University, Yuxi, China

**Keywords:** ecological management, host plant preference, push-pull strategy, trap plant, whitefly

## Abstract

**Introduction:**

This study aimed to identify promising trap plants for *Bemisia tabaci* among five host plants by integrating laboratory host preference and host suitability with semi-field population suppression under protected tomato cultivation.

**Methods:**

Laboratory assays assessed adult settling and oviposition preference and the survival and development of *B. tabaci* on the five plants. A greenhouse trial then evaluated whether planting these plants around protected tomato reduced *B. tabaci* densities on the main crop.

**Results:**

55.33% of female and 54.17% of male *B. tabaci* adults chose *Smallanthus sonchifolius*, whereas less than 10% of adults chose *Phaseolus vulgaris* and *Nicotiana tabacum*. The number of eggs laid by *B. tabaci* adults on *S. sonchifolius* reached 260.30 per plant, which was significantly higher than those on other plants. Considering the entire stage from egg to adult, the survival rate of *B. tabaci* on *S. sonchifolius* was highest (53.33%); and lowest on *N. tabacum* (23.83%). The development time of *B. tabaci* egg to nymph was longest on *N. tabacum* (33.45 days), and shortest on *S. sonchifolius* (21.78 days). The lifespan of adults was longest on *S. sonchifolius* (24.03 days), and shorter on *N. tabacum* (15.33 days). In a semi-field greenhouse trial, planting *S. sonchifolius* as a trap plant significantly reduced *B. tabaci* population on tomato plants under protected cultivation, with adult densities below 400 individuals per 100 cm^2^ leaf and nymph densities below 550 individuals per 100 cm^2^ leaf.

**Discussion:**

In summary, *B. tabaci* showed a strong settling and oviposition preference for *S. sonchifolius*, and this plant may have potential as an attractive trap plant for ecological management of *B. tabaci*, provided that *B. tabaci* on the trap plants is controlled promptly to prevent the plants from becoming a population source.

## 1 Introduction

The whitefly, *Bemisia tabaci* (Hemiptera: Aleyrodidae), is a globally destructive pest, widely distributed in over 90 countries and regions (Brown et al., 1995; Oliveira et al., 2001). This pest exhibits strong adaptability and invasive capabilities towards a wide range of plants, with host plants including tomato [*Solanum lycopersicum* L. (Solanales: Solanaceae)], cucumber [*Cucumis sativus* L. (Cucurbitales: Cucurbitaceae)], pepper [*Capsicum annuum* L. (Solanales: Solanaceae)], cotton [*Gossypium hirsutum* L. (Malvales: Malvaceae)], tobacco [*Nicotiana tabacum* L. (Solanales: Solanaceae)], and hollyhock [*Alcea rosea* L. (Malvales: Malvaceae)], among more than 1000 species of vegetables, economic crops, and ornamental plants (Dunn et al., 2024; Kedar et al., 2024). *B. tabaci* feeds directly on plant sap through its piercing-sucking mouthparts, causing physiological abnormalities in plants (Efekemo et al., 2024). The honeydew it excretes can induce sooty mold, affecting photosynthesis and reducing the quality of crops (Alvarez et al., 2024). Additionally, it serves as a vector for more than 300 plant viruses, such as tomato yellow leaf curl virus (TYLCV), tomato chlorosis virus (ToCV), and cucurbit chlorotic yellows virus (CCYV), leading to the occurrence of plant viral diseases. In recent years, outbreaks of tomato chlorosis virus (ToCV) and cucurbit chlorotic yellows virus (CCYV) in greenhouse vegetables in various regions have caused significant losses (Polston et al., 2014; Chu and Zhang, 2018; Xia et al., 2021; Thompson et al., 2024).

Trap plants, also known as lure plants, function by strategically positioning them in the field to attract target pests for feeding or oviposition, thereby reducing the pest population density on the main crop and minimizing the damage to the main crop. The cultivation of trap plants is a common biological and agricultural control method for managing agricultural pests, with a history of over a century (Liu et al., 2022; Gao et al., 2024; Qu et al., 2024). In recent years, numerous studies have been conducted domestically and internationally to screen and utilize trap plants for controlling pests that harm main crops. Hannunen (2003) investigated the relationship between different trap plant arrangements and the *Lygus rugulipennis* Poppius (Hemiptera: Miridae), and found that even a small area of trap plants could significantly reduce the pest density on the main crop. Hokkanen et al. (1986) discovered that Chinese cabbage and oilseed rape, used as trap crops, could effectively attract the *Meligethes aeneus* Fabricius (Coleoptera: Nitidulidae) from cauliflower in southern Finland. Gao et al. (2019) found that mint has a good lure effect on whiteflies, and intercropping mint with peppers can effectively control whiteflies on peppers fields. Therefore, exploring trap plant species that are strongly preferred by whiteflies for settling and oviposition plays a positive role in the ecological management of whiteflies.

During the initial investigation of the population distribution and host plant species of *B. tabaci* in Yunnan Province, China, we observed significant differences in the population numbers of adult and nymph whiteflies on different host plants. Notably, the populations on plants such as yacon [*Smallanthus sonchifolius* (Poepp. & Endl.) H.Rob. (Asterales: Asteraceae)], kidney bean [*Phaseolus vulgaris* L. (Fabales: Fabaceae)], tobacco (*N. tabacum*), hollyhock (*A. rosea*), and zucchini [*Cucurbita pepo* L. (Cucurbitales: Cucurbitaceae)] were significantly higher than on other plants (unpublished data). This suggests that *B. tabaci* prefers to feed and oviposit on these particular plants. Based on these findings, we hypothesize that *S. sonchifolius*, *P. vulgaris*, *N. tabacum*, *A. rosea*, and *C. pepo* have the potential to serve as trap plants for *B. tabaci*. The overarching objective of this study was to identify promising trap plants for *B. tabaci* among these five host plants by integrating laboratory host preference and host suitability with semi-field population suppression under protected tomato cultivation. To address this objective, we (i) assessed adult settling and oviposition preference among the five plants, (ii) compared survival and developmental performance on these plants, and (iii) evaluated whether planting them around protected tomato plants reduced *B. tabaci* densities on the main crop under greenhouse conditions. The results of this study will provide scientific evidence and guidance for the ecological management of *B. tabaci* and the improvement of the “push-pull” strategy theory.

## 2 Materials and methods

### 2.1 Insects and plants

The tested insect was *B. tabaci* Mediterranean (MED), this *B. tabaci* population was provided by the Yunnan Academy of Agricultural Sciences in Kunming, China. and was reared indoors earlier with tomato cv. ‘Zhongyan TV1’ (*S. lycopersicum*) as the host plant. These insects were kept in an artificial climate chamber (Shanghai Boxun Industrial Co., Ltd., BIC-300) for rearing, which was maintained at 25 °C temperature, 60–70% relative humidity (RH), 60% light intensity, and a photoperiod of 14 h:10 h light: dark cycle.

The trap plants used were yacon cv. ‘Huangxin’ (*S. sonchifolius*), kidney bean cv. ‘Honghuabaijia’ (*P. vulgaris*), tobacco cv. ‘Yunyan 87’ (*N. tabacum*), hollyhock cv. ‘Yizhanghong’ (*A. rosea*), and zucchini cv. ‘Lvbao’ (*C. pepo*). These plants were individually potted in a greenhouse using pots with a radius of 27 cm and a height of 18 cm. They were allowed to grow until the fourth leaf stage before being used for the experiments. For the validation of attraction effects in the field, the main crop was tomato cv. ‘Zhongyan TV1’ (*S. lycopersicum*), which was also individually potted in a greenhouse using pots with the same dimensions.

### 2.2 Behavioral attraction: settling, and oviposition preference of *Bemisia tabaci* among different trap plants

Five potted host plants with similar growth stages and leaf areas were placed around the inner perimeter of a test cage (base radius 80 cm, height 100 cm), one pot per species. Positions were randomized and re-randomized for each of three independent replicates. Plastic wrap was laid on the soil surface to prevent adults from falling into the soil. For each replicate, 200 female and male *B. tabaci* adults were starved for 0.5 h in a container (diameter 10 cm, height 6 cm), released from the cage center, and allowed to disperse freely. Adults on each plant were recorded at 6, 12, 24, and 48 h after release. Adults on cage walls, the container, or other non-plant surfaces were recorded as non-choosers. At 48 h, all leaves were removed and examined under a stereomicroscope (OLYMPUS SZ51) to count eggs on leaf undersides (Omondi et al., 2005; Yan et al., 2020; Wan et al., 2024). The assay was repeated three times with new plants and new insect cohorts under 25 ± 2 °C, 60–70% RH, and 4,700 lx; LED growth lights were evenly distributed above the cage to avoid phototaxis bias. Temperature, RH, and photoperiod were monitored daily.

### 2.3 Host suitability of different trap plants for *B. tabaci*: survival and developmental time

Observation of the survival rate of *Bemisia tabaci* on different trap plants

Ten pairs of *B. tabaci* adults were confined in micro-cages (diameter 3 cm, height 1 cm) on leaf undersides for 24 h to lay eggs. Micro-cages were randomly assigned to leaf and canopy positions. After removing adults, eggs were examined under a stereomicroscope (OLYMPUS SZ51); 40 eggs were retained per micro-cage, with five micro-cages and 200 eggs per plant species. Observations were made every 24 h to record nymphal development and survival until emergence or death (Iida et al., 2009; Mugerwa et al., 2019).

Observation of the developmental time of *Bemisia tabaci* on different trap plants

Five pairs of adults were confined in micro-cages on leaf undersides for 24 h. After removing adults, eggs were examined under a stereomicroscope, and excess eggs were removed to leave one egg per micro-cage. Eggs and nymphs were observed every 24 h until adult emergence, and the duration of each stage was recorded (González-Zamora and Moreno, 2011; Guo et al., 2013; Zhu et al., 2023). Thirty individuals were observed per plant species.

### 2.4 Semi-field evaluation of population suppression on protected tomato under greenhouse conditions

The trap crop experiment was conducted under protected cultivation in greenhouses in Yiliang County, Kunming City, China (24°47′56.180″ N, 103°09′32.612″ E). A completely randomized design was used. Each treatment, including the no-trap control, was established in a separate greenhouse compartment, with three independent greenhouse compartments (replicates) per treatment; thus, six treatments corresponded to 18 compartments. Treatments were randomly assigned to compartments. In each compartment, 150 tomato plants were planted in a 20 m × 6 m greenhouse in a rectangular pattern, with 30 plants per row and five rows, spaced 25 cm within rows and 80 cm between rows. At tomato transplanting, trap plants were placed as follows: 10 plants (2 rows × 5 plants) along each short side and 20 plants (2 rows × 10 plants) along each long side, totaling 60 trap plants per compartment. The control compartments had no trap plants. A certain number of *B. tabaci* adults were artificially released into each compartment to standardize the initial population. Ten days after tomato transplantation, 1,000 pairs of *B. tabaci* adults (female:male = 1:1) were released into each compartment, and populations on tomato and trap plants were surveyed every 10 days thereafter. For tomato, a five-point sampling method was used, with two plants randomly selected per point; three top leaves per plant were examined for adults and three middle-lower leaves for high-instar nymphs. For trap plants, ten top leaves per side were examined for adults and ten middle-lower leaves per side for nymphs. Leaf area was measured with a leaf area meter (SN-3001-YMJ) (Yang et al., 2022; Lun et al., 2024; Gao et al., 2024). Each greenhouse compartment had insect-proof mesh and natural ventilation; temperature and RH were recorded daily. No insecticides were applied, and water and fertilizer management were uniform across compartments.

### 2.5 Data analysis

Data were first tested for normality using the Shapiro–Wilk test and for homogeneity of variance using Bartlett’s test. For non-repeated data, including host preference/settling responses and developmental time, one-way ANOVA followed by least significant difference (LSD) analysis was used to compare the different trap plant groups, with the significance threshold set at *P* = 0.05. For the repeated population fluctuation data (adult and nymph densities recorded every 10 days), linear mixed models (LMMs) were used. Two separate datasets were analyzed. For the main crop (tomato), six treatments were compared: five trap plant species plus a no-trap control, giving 18 greenhouse compartments in total. For the trap plants, only the five trap plant species were compared, giving 15 greenhouse compartments, because the control compartments contained no trap plants. In both models, treatment, sampling date, and their interaction (treatment × sampling date) were included as fixed effects, and greenhouse compartment was included as a random effect to account for repeated measures on the same compartment over time. The repeated covariance structure was specified as first-order autoregressive (AR(1)). Models were fitted using restricted maximum likelihood (REML), and denominator degrees of freedom were estimated using the Satterthwaite approximation. When the treatment × sampling date interaction was significant, simple-effect analyses were performed to compare treatments within each sampling date, with Bonferroni adjustment for multiple comparisons. Each greenhouse compartment was treated as the experimental unit (n = 3 per treatment; 18 compartments for the main crop analysis and 15 compartments for the trap plant analysis). All analyses were performed using SPSS 20.0, and figures were created using Origin 2021.

## 3 Results

### 3.1 Behavioral attraction: settling and oviposition preference of *B. tabaci* among trap plants

The proportion of adults that settled on plants increased over time, from 62.50% (female) and 61.33% (male) at 6 h to 93.83% (female) and 96.18% (male) at 48 h. At 6 h, the highest proportions settled on *S. sonchifolius* (20.83% female, 15.83% male) and *C. pepo* (17.33% female, 16.17% male), while *N. tabacum* was least chosen (4.67% female, 4.00% male) (female: *F*_5,17_ = 60.666, *P* < 0.001; male: *F*_5,17_ = 218.543, *P* < 0.001). At 12 h, *S. sonchifolius* was the most chosen (31.67% female, 31.50% male), and *N. tabacum* the least (5.67% female, 5.17% male) (female: *F*_5,17_ = 115.936, *P* < 0.001; male: *F*_5,17_ = 153.400, *P* < 0.001). At 24 h, *S. sonchifolius* remained the most chosen (38.67% for both sexes), and *N. tabacum* the least (4.67% female, 5.50% male) (female: *F*_5,17_ = 50.122, *P* < 0.001; male: *F*_5,17_ = 84.922, *P* < 0.001). At 48 h, *S. sonchifolius* was chosen by 55.33% of females and 54.17% of males, while *P. vulgaris* (6.33% female, 9.83% male) and *N. tabacum* (5.00% female, 7.67% male) were least chosen (female: *F*_5,17_ = 73.291, *P* < 0.001; male: *F*_5,17_ = 262.943, *P* < 0.001) (**Figure 1**).

**Figure 1:**
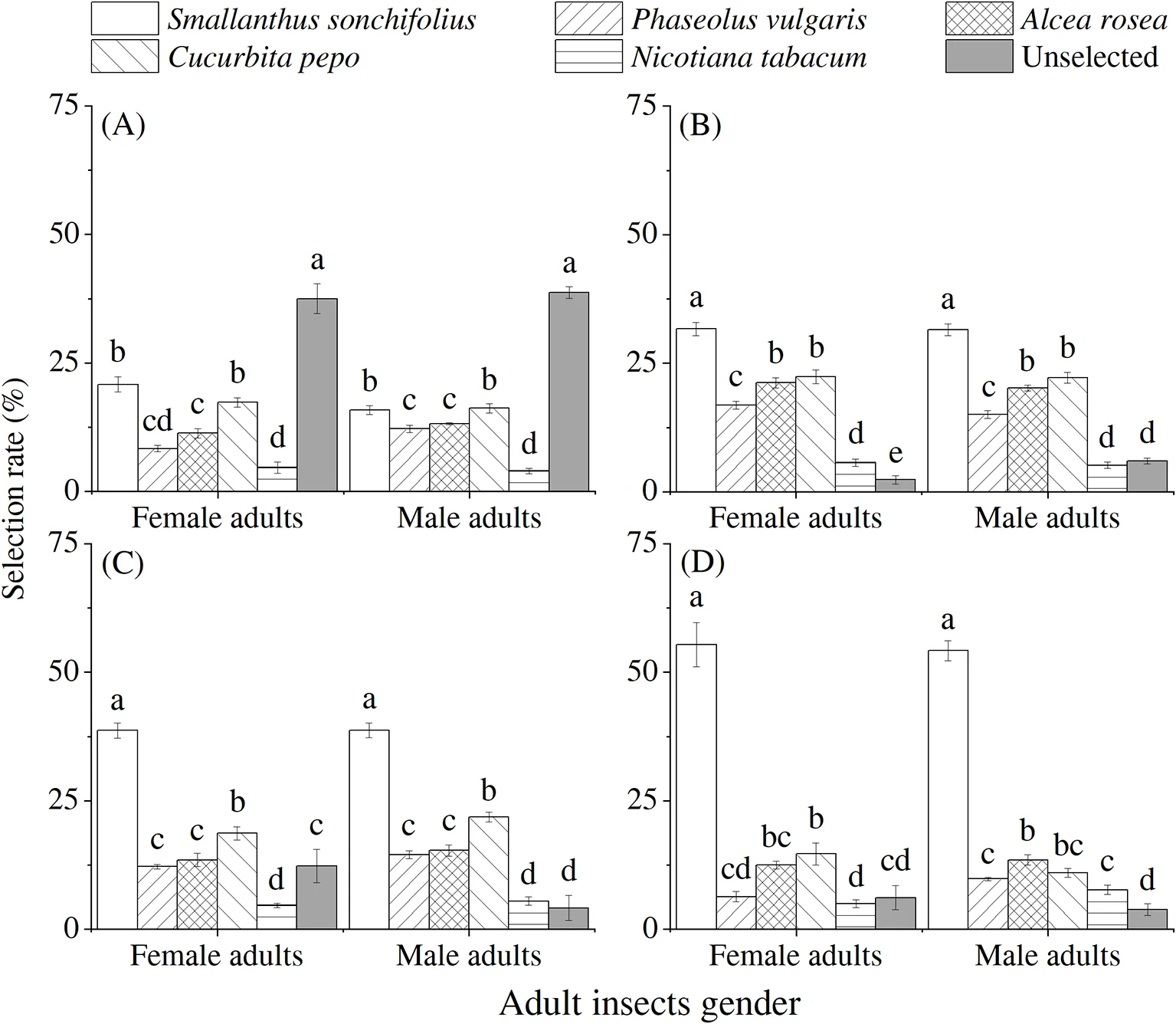
The host selection (mean ± standard error) of *Bemisia tabaci* adults on different trap plants. **(A)** Selection rates at 6 hours after release; **(B)** selection rates at 12 hours after release; **(C)** selection rates at 24 hours after release; **(D)** selection rates at 48 hours after release. The different lowercase letters indicate significant differences at the 0.05 level according to the least significant differences analysis.

The oviposition preference of *B. tabaci* adults for different trap plants was significantly different. At 48 hours after release, the number of eggs laid by *B. tabaci* on *S. sonchifolius* plant was significantly higher than that on other plants, reaching 260.30 ind. per plant. In contrast, the number of eggs laid by *B. tabaci* on other species plants was relatively low, at only 32.00 ind. per plant on *P. vulgaris* plant, 57.00 ind. per plant on *A. rosea* plant, 71.67 ind. per plant on *C. pepo* plant, and 22.67 ind. per plant on *N. tabacum* plant, respectively (*F*_4, 14_ = 148.483, *P* < 0.001) (**Figure 2**).

**Figure 2:**
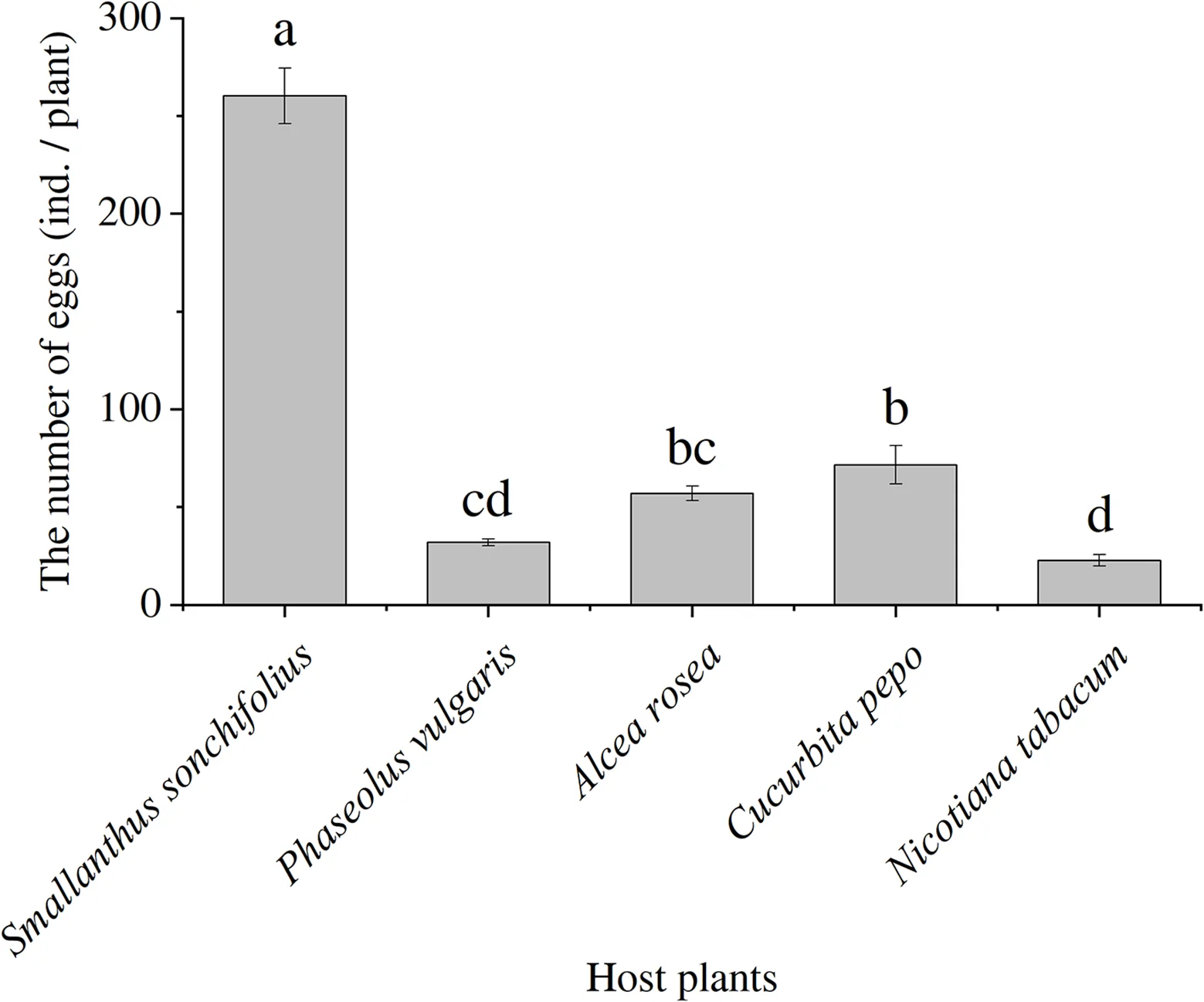
The number (mean ± standard error) of eggs laid by *Bemisia tabaci* adults on different trap plants. The different lowercase letters indicate significant differences at the 0.05 level according to the least significant differences analysis.

### 3.2 Host suitability: survival and developmental performance of *B. tabaci* on trap plants

Survival rates differed significantly among developmental stages and trap plants (**Table 1**). For all plants, survival from the 2nd to 3rd instar, 3rd to 4th instar, and 4th instar to adult was around 90%, significantly higher than egg to 1st instar and 1st to 2nd instar (*S. sonchifolius*: *F*_5,17_ = 64.296; *P. vulgaris*: *F*_5,17_ = 103.253; *A. rosea*: *F*_5,17_ = 119.860; *C. pepo*: *F*_5,17_ = 133.293; *N. tabacum*: *F*_5,17_ = 51.373; all *P* < 0.001). Among plants, *S. sonchifolius* had the highest egg-to-1st-instar survival (83.33%) and *N. tabacum* the lowest (57.83%) (*F*_4,14_ = 15.679, *P* < 0.001). For 1st to 2nd instar, survival was around 80% on *S. sonchifolius*, *P. vulgaris*, and *C. pepo*, significantly higher than on *N. tabacum* (69.00%) (*F*_4,14_ = 3.455, *P* = 0.051). For 2nd to 3rd instar, *S. sonchifolius* had the highest survival (92.53%), significantly higher than *N. tabacum* (85.86%) (*F*_4,14_ = 2.141, *P* = 0.150). For 3rd to 4th instar, survival exceeded 90% on *S. sonchifolius*, *P. vulgaris*, and *C. pepo*, significantly higher than *N. tabacum* (84.26%) (*F*_4,14_ = 4.251, *P* = 0.029). For 4th instar to adult, no significant differences were found (all around 90%) (*F*_4,14_ = 1.571, *P* = 0.256). Egg-to-adult survival was highest on *S. sonchifolius* (53.33%) and lowest on *N. tabacum* (23.83%) (*F*_4,14_ = 44.195, *P* < 0.001) (**Table 1**).

**Table 1:** The survival rate (mean ± standard error) of *Bemisia tabaci* on different trap plants.

Trap plants	Survival rate (%)					
	Egg-1st instar nymph	1st-2nd instar nymph	2nd-3rd instar nymph	3rd-4th instar nymph	4th instar nymph- adult	Egg-adult
*Smallanthus sonchifolius*	83.33 ± 2.05aB	83.68 ± 1.98aB	92.53 ± 2.21aA	92.31 ± 0.90aA	89.75 ± 2.49aA	53.33 ± 0.73aC
*Phaseolus vulgaris*	67.67 ± 2.49bC	78.46 ± 1.71aB	90.09 ± 1.08abA	91.13 ± 2.49aA	88.55 ± 0.81aA	38.67 ± 2.59bcD
*Alcea rosea*	67.33 ± 2.24bC	76.75 ± 0.44abB	87.50 ± 2.16abA	88.97 ± 2.24abA	91.39 ± 2.47aA	36.67 ± 0.73cD
*Cucurbita pepo*	72.17 ± 2.42bC	79.97 ± 1.81aB	87.80 ± 1.52abA	92.10 ± 0.51aA	91.80 ± 0.84aA	42.83 ± 1.86bD
*Nicotiana tabacum*	57.83 ± 2.46cC	69.00 ± 5.69bB	85.86 ± 1.65bA	84.26 ± 0.90bA	83.32 ± 4.84aA	23.83 ± 1.30dD

The different lowercase letters indicate significant differences at the 0.05 level according to the least significant differences analysis at the same developmental stage; the different capital letters indicate significant differences at the 0.05 level according to the least significant differences analysis at the same trap plant.

Developmental time also differed significantly among plants (**Table 2**). *N. tabacum* had the longest duration for egg (7.57 d), 1st instar (6.47 d), 2nd instar (5.97 d), 3rd instar (5.77 d), and 4th instar (7.67 d), while *S. sonchifolius* had the shortest egg duration (4.27 d) (egg: *F*_4,149_ = 82.676; 1st instar: *F*_4,149_ = 53.363; 2nd instar: *F*_4,149_ = 77.211; 3rd instar: *F*_4,149_ = 87.613; 4th instar: *F*_4,149_ = 44.617; all *P* < 0.001). Adult longevity was longest on *S. sonchifolius* (24.03 d) and shortest on *C. pepo* (13.77 d) (*F*_4,149_ = 55.071, *P* < 0.001).

**Table 2:** The development time (mean ± standard error) of *Bemisia tabaci* on different trap plants.

Trap plants	Development time (days)					
	Egg	1st instar nymph	2nd instar nymph	3rd instar nymph	4th instar nymph	Adult
*Smallanthus sonchifolius*	4.27 ± 0.17 e	4.10 ± 0.15 b	3.47 ± 0.14 b	3.87 ± 0.12 b	6.07 ± 0.14 b	24.03 ± 0.58 a
*Phaseolus vulgaris*	6.10 ± 0.12 b	4.03 ± 0.13 b	3.23 ± 0.12 b	3.10 ± 0.13 c	5.83 ± 0.15 b	15.93 ± 0.39 c
*Alcea rosea*	5.57 ± 0.13 c	4.17 ± 0.17 b	3.37 ± 0.12 b	3.17 ± 0.11 c	6.17 ± 0.14 b	17.70 ± 0.53 b
*Cucurbita pepo*	4.97 ± 0.12 d	3.93 ± 0.14 b	3.43 ± 0.15 b	3.23 ± 0.11 c	4.93 ± 0.14 c	13.77 ± 0.60 d
*Nicotiana tabacum*	7.57 ± 0.14 a	6.47 ± 0.15 a	5.97 ± 0.12 a	5.77 ± 0.13 a	7.67 ± 0.17 a	15.33 ± 0.56 c

The different lowercase letters indicate significant differences at the 0.05 level according to the least significant differences analysis at the same developmental stage.

### 3.3 Population suppression: semi-field effects of trap plants on *B. tabaci* densities on protected tomato

Linear mixed models were used to analyze the repeated population fluctuation data. The tests of fixed effects are summarized in **Table 3**. Adult and nymph densities were analyzed separately for the main crop (tomato) and for the trap plants. For tomato, six treatments were compared (five trap plant species plus a no-trap control; 18 greenhouse compartments). For trap plants, only the five trap plant species were compared (15 greenhouse compartments), because the control compartments contained no trap plants.

**Table 3:** Tests of fixed effects for adult and nymph densities of *Bemisia tabaci* on protected tomato plants under different trap plant treatments.

Plant category	Response variable	Effect	*F*	*df*1	*df*2	*P*
Main crop (tomato)	Adult density	Treatment	208.62	5	11.98	< 0.05
		Sampling date	466.87	13	79.07	< 0.05
		Treatment × Sampling date	58.52	65	79.07	< 0.05
	Nymph density	Treatment	491.67	5	12.00	< 0.05
		Sampling date	2278.80	13	83.26	< 0.05
		Treatment × Sampling date	136.50	65	83.26	< 0.05
Trap plants	Adult density	Treatment	28.40	4	10.03	< 0.05
		Sampling date	51.71	13	68.97	< 0.05
		Treatment × Sampling date	7.98	52	68.97	< 0.05
	Nymph density	Treatment	70.15	4	10.00	< 0.05
		Sampling date	310.68	13	71.06	< 0.05
		Treatment × Sampling date	15.38	52	71.06	< 0.05

Adult and nymph densities were analyzed using linear mixed models. Treatment, sampling date, and their interaction (treatment × sampling date) were included as fixed effects. Greenhouse compartment was included as a random effect, and the repeated covariance structure was first-order autoregressive [AR(1)]. Denominator degrees of freedom were estimated using the Satterthwaite approximation. *df*1, numerator degrees of freedom; *df*2, denominator degrees of freedom. *P* < 0.05 indicates a significant effect.

For adult density on tomato, the linear mixed model showed significant effects of treatment (*F*_5, 11.98_ = 208.62, *P* < 0.05), sampling date (*F*_13, 79.07_ = 466.87, *P* < 0.05), and their interaction (*F*_65, 79.07_ = 58.52, *P* < 0.05) (**Table 3**). Because the treatment × sampling date interaction was significant, simple-effect analyses were performed within each sampling date. When *S. sonchifolius* was used as the trap plant, adult density on tomato remained at approximately 300.00–400.00 individuals per 100 cm² leaf from early August to mid-October and was significantly lower than that in the control and the *N. tabacum* treatment on most sampling dates (*P* < 0.05). In contrast, the control and *N. tabacum* treatments showed the highest peaks (1038.89 and 1271.83 individuals per 100 cm^2^ leaf, respectively), whereas the other trap plant treatments showed intermediate peaks (**Figure 3A**).

**Figure 3:**
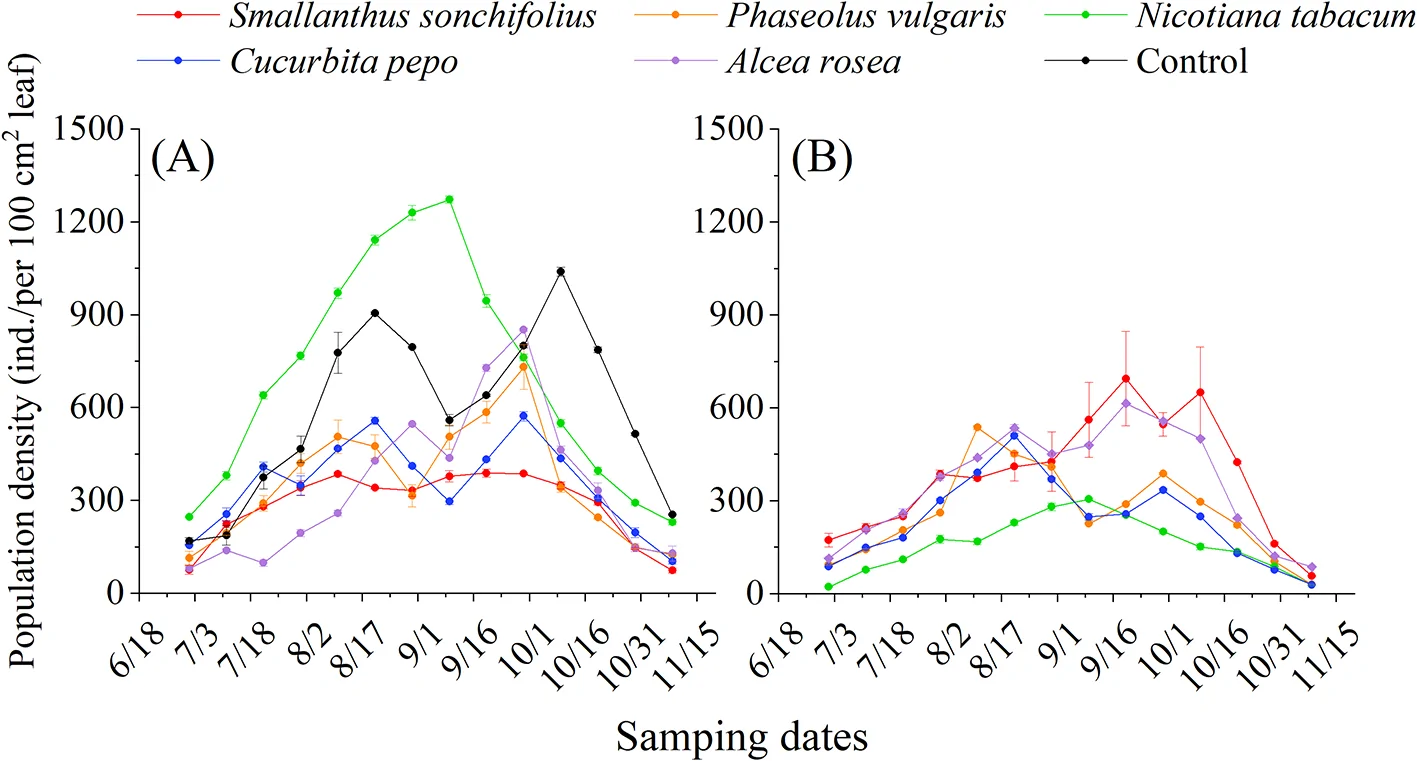
Population fluctuation of *Bemisia tabaci* adults (mean ± standard error) on protected tomato (main crop) and trap plants under greenhouse conditions. **(A)** Main crop (tomato), **(B)** trap plants.

For adult density on trap plants, the linear mixed model included only the five trap plant species (15 compartments) because the control compartments had no trap plants. Treatment (*F*_4, 10.03_ = 28.40, *P* < 0.05), sampling date (*F*_13, 68.97_ = 51.71, *P* < 0.05), and their interaction (*F*_52, 68.97_ = 7.98, *P* < 0.05) were all significant (**Table 3**). Simple-effect analyses showed that adult density on *S. sonchifolius* peaked in mid- to late September (693.81 individuals per 100 cm^2^ leaf) and again in early October (649.94 individuals per 100 cm² leaf), whereas *P. vulgaris* and *C. pepo* peaked earlier, from early to mid-August (*P. vulgaris*: 536.29; *C. pepo*: 535.03 individuals per 100 cm^2^ leaf) and again in late September (*P. vulgaris*: 387.14; *C. pepo*: 613.99 individuals per 100 cm^2^ leaf). *A. rosea* peaked in late August (509.42 individuals per 100 cm^2^ leaf) and at the end of September (333.38 individuals per 100 cm^2^ leaf), and *N. tabacum* peaked in early September (305.10 individuals per 100 cm^2^ leaf) (**Figure 3B**).

For nymph density on tomato, treatment (*F*_5, 12.00_ = 491.67, *P* < 0.05), sampling date (*F*_13, 83.26_ = 2278.80, *P* < 0.05), and their interaction (*F*_65, 83.26_ = 136.50, *P* < 0.05) were also significant (**Table 3**). Simple-effect analyses showed that when *S. sonchifolius* was used as the trap plant, nymph density on tomato was maintained below approximately 550.00 individuals per 100 cm^2^ leaf and was significantly lower than that in the control on most sampling dates (*P* < 0.05). The control and *N. tabacum* treatments reached the highest peaks (1530.52 and 905.19 individuals per 100 cm^2^ leaf, respectively), while the other trap plant treatments showed intermediate levels (**Figure 4A**).

**Figure 4:**
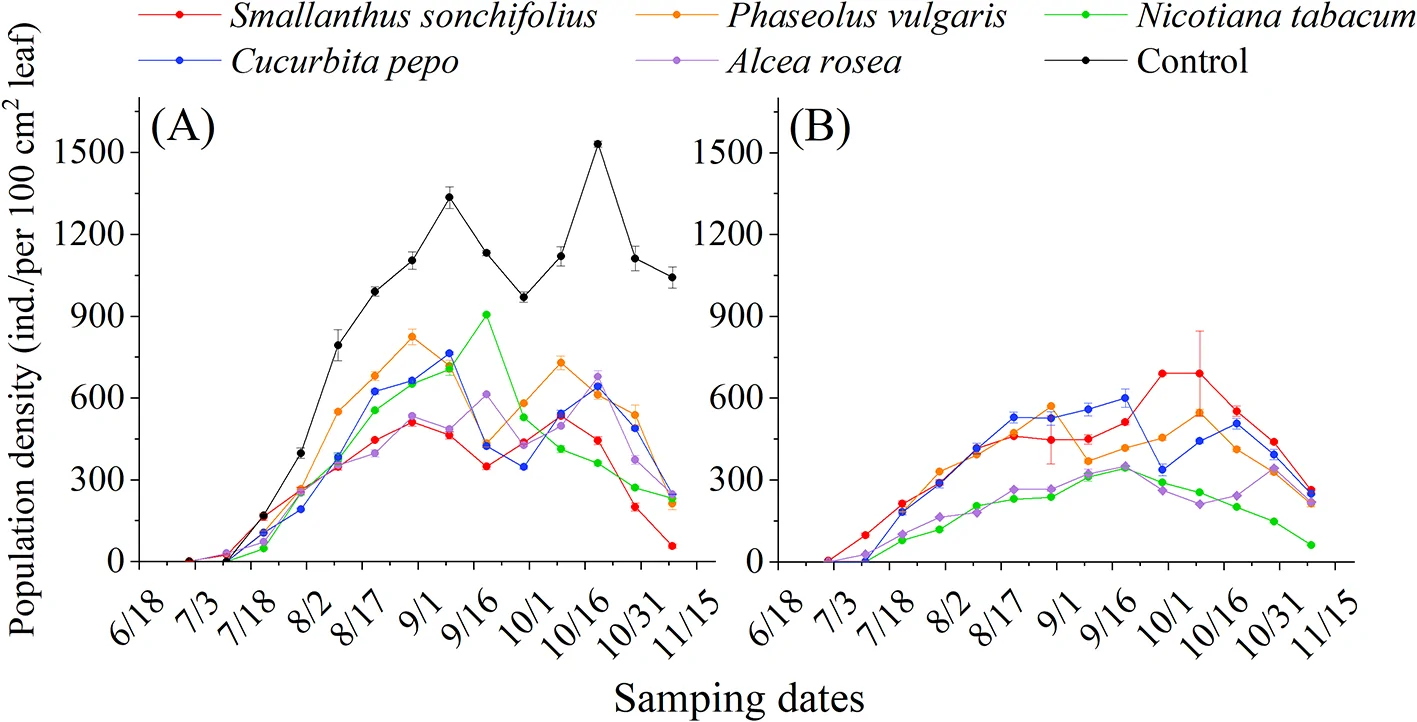
Population fluctuation of *Bemisia tabaci* nymphs (mean ± standard error) on protected tomato (main crop) and trap plants under greenhouse conditions. **(A)** Main crop (tomato), **(B)** trap plants.

For nymph density on trap plants, treatment (*F*_4, 10.00_ = 70.15, *P* < 0.05), sampling date (*F*_13, 71.06_ = 310.68, *P* < 0.05), and their interaction (*F*_52, 71.06_ = 15.38, *P* < 0.05) were also significant (**Table 3**). Simple-effect analyses showed that nymph density on *S. sonchifolius* peaked at the end of September (690.04 individuals per 100 cm^2^ leaf), while *P. vulgaris* peaked at the end of August (569.68 individuals per 100 cm^2^ leaf) and again in early October (546.66 individuals per 100 cm^2^ leaf). *C. pepo* peaked in mid- to late September (350.92 individuals per 100 cm^2^ leaf) and at the end of October (342.90 individuals per 100 cm^2^ leaf). *N. tabacum* and *A. rosea* both peaked in mid- to late September (*N. tabacum*: 343.23; *A. rosea*: 600.18 individuals per 100 cm^2^ leaf), with *A. rosea* showing a second peak in mid- to late October (507.20 individuals per 100 cm^2^ leaf) (**Figure 4B**).

Overall, the linear mixed models showed that treatment, sampling date, and their interaction significantly affected adult and nymph densities of *B. tabaci* on both tomato and trap plants (**Table 3**). Simple-effect analyses indicated that *S. sonchifolius* consistently maintained lower *B. tabaci* densities on tomato than the control and most other trap plant treatments, particularly for adults and nymphs during the main infestation period.

## 4 Discussion

After release, more *B. tabaci* adults settled on *S. sonchifolius* and more eggs were laid on this plant than on the other plants, indicating a strong settling and oviposition preference for *S. sonchifolius*. Pyke et al. (1987) proposed the “Push-Pull” strategy, which involves manipulating the behavior of pests and their natural enemies to control their distribution and density in the field, thereby reducing the damage of pests to main crops. The “Push-Pull” strategy consists of two parts: “push” and “pull”. Through crop planting arrangements, the protected economic crops are made unattractive or unsuitable for pest growth and development (push), while pests are lured to another more attractive crop with lower economic value (pull), and then concentrated for management. The “push” components mainly include visual interference, non-host volatiles, anti-aggregation pheromones, and alarm pheromones; the “pull” components mainly include visual attraction, host volatile, and aggregation pheromones (Cook et al., 2007). The focus of this study is on “pulling” the *B. tabaci* population to the trap plants for concentrated management. Many researchers have used crop arrangements or developed repellents and attractants to implement the “Push-Pull” strategy in agricultural production. Cook et al. (2004) found that planting *Brassica rapa* L. (Brassicales: Brassicaceae) on the edges of *Brassica napus* fields can reduce the species abundance of *Meligethes aeneus* Fabricius (Coleoptera: Nitidulidae) and *Ceutorhynchus assimilis* Paykull (Coleoptera: Curculionidae), which were two major pests in *B. napus* fields. In Australia, people plant trap plants such as *Cajanus cajan* (L.) Huth (Fabales: Fabaceae) and *Zea mays* L. (Poales: Poaceae) on the edges of cotton fields to control the *Helicoverpa armigera* Hübner (Lepidoptera: Noctuidae), greatly reducing the damage of *H. armigera* to cotton (Duraimurugan and Regupathy, 2005; Cook et al., 2007). Yeboah et al. (2021) planted *Pennisetum purpureum* Schumach. (Poales: Poaceae) as a trap plant to control the *Mythimna separata* Walker (Lepidoptera: Noctuidae) in *Z. mays* fields, achieving significant control effects. This study found that *B. tabaci* showed a strong settling and oviposition preference for *S. sonchifolius*, and this plant can be planted as a trap plant on the edges of main crop fields to “pull” the *B. tabaci* population for concentrated management, serving as an ecological management approach for the control of *B. tabaci*.

Overall, the survival rate of *B. tabaci* population on *S. sonchifolius* was the highest, while on *N. tabacum* it was the lowest and the developmental time was the longest. Although the host plants of *B. tabaci* exceeds 1,000 species, different host plants also have different effects on its growth and development. On the one hand, the nutrients provided by different host plants to *B. tabaci* vary. it directly affects the size of *B. tabaci* individuals (Lin et al., 2003; Frank, 2009). In terms of plant characteristics, *S. sonchifolius* has larger leaves and a longer growth period, and its leaves contain a variety of compounds such as sugars, amino acids, and mineral elements (Fernandez et al., 2006; Žiarovská et al., 2019), which provide better nutritional conditions for the development of *B. tabaci* nymphs. This may be one of the reasons why *S. sonchifolius* was conducive to the survival and development of *B. tabaci*. If host plants can provide *B. tabaci* with a good habitat and feeding conditions, *B. tabaci* will preferentially choose host plants that are more conducive to the development of their offspring (Zhou et al., 2008). Zhao et al. (2018) found that the developmental duration of *B. tabaci* on *S. sonchifolius* and *N. tabacum* was significantly different, with the developmental duration on *S. sonchifolius* being about 1.6 days shorter than that on *N. tabacum*. Zhu et al. (2023) found that the developmental duration of *B. tabaci* on *S. sonchifolius* was the shortest, the individual fertility was the highest, and the net reproductive rate was the highest; while on *N. tabacum*, the developmental duration of *B. tabaci* was prolonged, the individual fertility decreased, and the intrinsic rate of increase and finite rate of increase decreased. The research results are consistent with this study. On the other hand, different plants have different resistance to pests. *B. tabaci* infestation can significantly activate the Salicylic Acid (SA) defense pathway in *N. tabacum* plants, and after 15 days of feeding, the level of salicylic acid is 1.40 times higher than that of the control (Xu et al., 2020). SA is one of the important hormones in plants, which can mediate the production of defense responses in plants to resist biotic and abiotic stresses (Shojaaddini, 2024; Zhu et al., 2024). When 2–3 nymphs cm^-^² infest *N. tabacum*, it can induce the resistance of *N. tabacum plant*, reducing the developmental rate of pests on the plant, prolonging the developmental duration, and decreasing the average number of eggs laid by a single female (Wang, 2023).

In addition to the direct application of trap plants, many researchers have also utilized extracts from these plants to attract target pests in the field. Most phytophagous insects in nature mainly rely on their sense of smell to locate and distinguish between host and non-host plants, using the chemical substances released by host plants to determine their flight direction and accurately find hosts for mating, oviposition, and avoiding predators, among other physiological activities (Lu et al., 2008; Kaupp, 2010). Chen (2021) measured the effects of three plant extracts (linalool, Z-3-hexenyl acetate, and nonanal) on the orientation behavior of *B. tabaci*, and the results indicated that linalool and Z-3-hexenyl acetate have a significant attraction to *B. tabaci*. The study by Ramírez-Godoy et al. (2018) showed that essential oil extracted from *Cocos nucifera* L. (Arecales: Arecaceae) had a significant trapping effect on the *Diaphorina citri* Kuwayama (Hemiptera: Psyllidae) and can be jointly developed with other insecticides as a lure for the *D. citri*. Qiu et al. (2018) found that at a high concentration essential oil extracted from *Dalbergia hainanensis* Merr. & Chun (Fabales: Fabaceae) showed a significant repellent effect on the *D. citri*, while at a low concentration, it exhibited a significant attraction effect. This indicates that the tendency of pests to odors is not fixed and may change with the variation of odor concentration. Therefore, in this study, the effective components and optimal concentrations of plants with good attraction effects on *B. tabaci* need further research.

An important question is whether *S. sonchifolius* should be considered a true trap plant or an attractive source. Unlike dead-end trap plants that attract pests but do not support their development, *S. sonchifolius* was both highly attractive and highly suitable for *B. tabaci*, as shown by the highest oviposition, highest survival, and shortest development time. Therefore, its ecological role cannot be defined solely by host suitability; it depends on whether the attracted whiteflies are subsequently controlled. Under a push-pull framework (Cook et al., 2007), a trap plant does not have to be a poor host; it can be a preferred host that concentrates pests away from the main crop, provided that the concentrated population is suppressed before it disperses. In our semi-field greenhouse experiment, planting *S. sonchifolius* around protected tomato significantly reduced *B. tabaci* adult and nymph densities on tomato compared with the control and other trap-plant treatments, indicating that it functioned as an effective attractive trap plant under the tested arrangement and sampling period. However, because *S. sonchifolius* supported high oviposition, high survival, and rapid development, unmanaged trap plants could become an attractive sink or even a population source, potentially increasing local *B. tabaci* populations and later spilling back to the main crop. We therefore recommend that *S. sonchifolius* be used with timely management of whiteflies on the trap plants, such as targeted insecticide application, pruning, or removal before adult emergence, and that trap-plant area, planting time, and control thresholds be optimized. Future studies should quantify source-sink fluctuation, adult dispersal between trap plants and tomato, and potential effects on natural enemies.

Several limitations should be acknowledged before this trap-plant strategy can be recommended as a complete IPM tool. First, we focused on *B. tabaci* population density and did not evaluate effects on natural enemies, such as predators and parasitoids. Trap plants could potentially conserve or attract natural enemies, but they could also disrupt biological control if not managed properly. Second, we did not measure tomato yield, fruit quality, or marketable yield, so the agronomic benefit of reduced whitefly density remains unquantified. Third, we did not assess virus incidence or transmission. Because *B. tabaci* is a vector of multiple viruses, a reduction in vector density does not necessarily translate into a proportional reduction in virus spread. Finally, it should be emphasized that our laboratory experiment measured host selection, settling, and oviposition preference, not olfactory orientation or chemical attraction. Adults released in the center of the cage could use visual and olfactory cues to select among plants. Therefore, the observed preference for *S. sonchifolius* cannot be attributed specifically to volatile chemical attraction. Future studies using Y-tube or four-arm olfactometers, electroantennography (EAG), and GC-MS volatile profiling are needed to identify whether specific volatile compounds mediate this preference and to determine the optimal attractive blend and concentration. Long-term, multi-season, multi-location trials integrating natural enemy monitoring, yield and quality assessment, virus incidence, and economic analysis are therefore required to evaluate the full potential and limitations of *S. sonchifolius* in IPM programs.

## 5 Conclusion

*S. sonchifolius* was strongly preferred by *B. tabaci* adults for settling and oviposition, and it provided a suitable host environment, supporting the highest survival rate and shortest development time of *B. tabaci* from egg to adult. Semi-field greenhouse trials confirmed that planting *S. sonchifolius* as a trap crop around protected tomato fields effectively reduced *B. tabaci* population density on the main crop under protected cultivation, with both adult and nymph counts maintained at low levels. However, because *S. sonchifolius* is also a suitable host, its role as a trap plant depends on timely management: without control of *B. tabaci* on the trap plants, it could become an attractive sink or population source. Collectively, these findings indicate that *S. sonchifolius* may have potential as an attractive trap plant within a push‐pull strategy for the ecological management of *B. tabaci* under protected cultivation, provided that trap-plant layout, planting time, and control thresholds are optimized and that whiteflies on the trap plants are controlled before adult emergence. This provides both theoretical support and practical guidance for sustainable integrated pest management programs.

## Data Availability

The raw data supporting the conclusions of this article will be made available by the authors, without undue reservation.
